# Stage-Specific Microbiota Transitions Throughout Black Soldier Fly Ontogeny

**DOI:** 10.1007/s00248-025-02691-1

**Published:** 2026-01-10

**Authors:** Thomas Klammsteiner, Carina D. Heussler, Katharina T. Stonig, Heribert Insam, Birgit C. Schlick-Steiner, Florian M. Steiner

**Affiliations:** 1 Department of Ecology, Universität Innsbruck, Technikerstr. 25, 6020 Innsbruck, Austria; 2 BioTreaT GmbH, Technikerstr. 21d, Innsbruck, 6020 Austria

**Keywords:** Attractants, Interkingdom communication, Circular economy, Insect farming, Microbial colonization, Insect immunity

## Abstract

The growing global population increases the demand for protein, while organic waste management has become more challenging. Alternative protein sources are essential to mitigate the environmental impact of food production. The black soldier fly (BSF; *Hermetia illucens*) has emerged as an alternative to traditional protein sources (e.g., soybean meal, fishmeal) due to its ability to convert diverse organic waste, addressing both issues simultaneously. This makes the BSF a promising candidate for industrial rearing, with its successful development closely tied to microbial influences on growth and behaviour, particularly bacterial influences on oviposition. In this study, we focus on the microbiota throughout insect development with a special focus on egg surface microbiota and their origin. We analysed the microbiota in the haemolymph and gut of larvae raised on sterilized and non-sterilized feed, pupal cell pulp, the wash of the ovipositor, eggs directly collected after oviposition, ovarian eggs, the empty female abdomen, eggs exposed to adult BSF, and surface-sterilized eggs. Our analysis revealed distinct bacterial community profiles across life stages, indicating a transition from larval dominance of Enterobacteriaceae to Burkholderiaceae on all analysed eggs. At the genus level, larval stages were characterized by *Morganella*, *Escherichia*, and *Proteus*, transitioning to less diverse communities in egg samples predominated by *Burkholderia-Caballeronia-Paraburkholderia*. Our study reveals that while predominant microbiota persist throughout all life stages, microbial community composition transforms progressively during maturation, particularly before oviposition. Understanding egg surface microbiota and the cues guiding oviposition has the potential to boost egg production and simplify mass harvesting of BSF larvae.

**Supplementary Information:**

The online version contains supplementary material available at 10.1007/s00248-025-02691-1.

## Introduction

To reduce the environmental impact of food production, it is central to consider alternative protein sources for animal feed, especially given the heavy environmental drawbacks associated with soybean and fishmeal. In South America, the area used for soybean cultivation more than doubled within the ten years until 2019, reaching approx. 55 million hectares [1]. Up to 99% of deforestation of tropical forests is driven by agriculture, with an estimated area loss of up to 8.8 million hectares per year [2]. Collectively, agrifood systems are responsible of 29.7% (more than 16 billion tons in CO_2_eq) of global greenhouse gas emissions [3]. At the same time, continued population growth and rising incomes have intensified the problem of food loss and waste, resulting in almost one fifth of agricultural land and water being dedicated to growing food that is ultimately lost or wasted [4]. Reduction fisheries, turning wild fish into fishmeal as feed for farmed fish, put increasing pressure on wild fish populations and climate change driven shortages in fish meal and oil production may lead to unpredictable price development [5]. The FAOs 2024 report on The State of World Fisheries and Aquaculture highlights that despite aquaculture has exceeded for the first time capture fisheries in the production of aquatic animals, overall production of aquatic animals is projected to reach 205 million tons by 2032 [6]. Insect biomass as an alternative source of animal protein has been reported to have a lower environmental impact than other sources of animal protein [7]. In the appropriate framework, insect farming emits less greenhouse gases, uses less land, and requires lower inputs of feed and water due to higher conversion efficiencies [7, 8].

In the last years, the black soldier fly (BSF), *Hermetia illucens* (Linnaeus, 1758), has been identified as a key player in substituting animal protein for animal feed production and for reducing the reliance on unsustainable soybean and fishmeal [7]. BSF larvae (BSFL) are high in protein (37–63% dry matter base) and fat (up to 49% dry matter base) and contain several micro-and macronutrients important for livestock health and development [9]. Under optimal conditions, the fly’s life cycle takes approx. 40–45 days, progressing through five developmental stages: egg, larva (with six instars including prepupal stage), pupa, and adult [10]. The larval stage is characterized by fast growth and five successive molts before transitioning to non-feeding pupation [11]. The larvae can digest a large variety of organic matter such as food waste, faecal sludge, manure, and agro-industrial by-products [12]. The undigested residues mixed with BSFL excrements find use as organic fertilizer, with the potential to substitute or replace mineral fertilizers [13, 14]. By converting organic wastes into nutrient-rich insect biomass suitable for feedstock production with organic fertilizer as the main process by-product, the BSF can contribute to circular economy goals [15]. For these and many more reasons, the BSF has become popular for industrial insect rearing. Key challenges frequently highlighted in this sector include the limited number of studies that go beyond the laboratory scale, inconsistent regulatory frameworks across countries, dim prospects for future approval of food waste as a substrate, and widely held predictions that insect protein will face significant difficulties competing with economically with established protein sources such as soybean and fishmeal [16]. However, as this novel industry is rapidly growing into more mature stages, it has become clear that a more profound and comprehensive understanding of the fly’s biology is needed to tackle challenges in upscaling production. Its successful development is closely tied to microbes that affect its growth and behaviour, such as bacteria that impact its oviposition. Despite extensive research on the gut microbiome, a knowledge gap persists in understanding microbial colonization across all stages, notably on the egg surface [10, 17, 18]. Successful large-scale rearing of BSF depends on a flourishing reproduction of adults.

Understanding microbial colonization patterns across BSF life stages is crucial for optimizing industrial production, as bacterial communities exhibit distinct dynamics during development [19]. Moreover, multiple studies demonstrated that BSF microbiota composition can vary significantly among colonies from different laboratories, where genetic background and rearing environment play central roles in shaping these differences [20, 21]. Global comparisons have shown distinct microbial communities in BSFL for geographically separated colonies [22]. Substrate properties and developmental stage contribute to microbiota variation, underlining the importance of standardized rearing conditions for reproducible results in BSF research [23]. Recent research has shown that in insects, transmission of microorganisms can occur through various routes, including vertical transmission from parent to offspring through egg surface colonization, which plays a central role in supporting early-life microbiomes [24, 25]. In BSF, bacterial communities undergo significant restructuring during metamorphosis, with Proteobacteria and Firmicutes typically dominating larval stages, while bacterial diversity and composition change notably during the transition from pupal to adult stages [19, 26, 27]. The time point and mechanism of microbial acquisition varies across insect species, with some exhibiting maternal inheritance through specialized transmission mechanisms such as egg surface inoculation or the deposition of symbiont-containing capsules [28, 29]. While microorganisms associated with substrates primarily influence larval gut microbiota composition and metabolism, the establishment of egg-associated bacterial communities represents a critical yet understudied aspect of BSF reproduction that may directly impact offspring viability and subsequent larval development [30].

In addition, microbes play a crucial role in shaping the reproductive performance of BSF [31]. The female adults oviposit single eggs in clutches (up to 900 eggs) close to decomposing organic matter and are attracted by conspecific eggs [18, 32]. Several studies showed that bacteria isolated from conspecific eggs attract gravid females, presumably by emissions of volatile organic compounds [18, 33–35]. Understanding the presence of microbes throughout different life stages of the BSF is central for optimizing industrial production, as a better understanding of microbe-host interactions, particularly interkingdom communication involved in oviposition regulation, can enhance egg production and increase the efficiency of larval mass rearing [18, 36, 37].

This study focused on investigating the bacterial communities throughout BSF life stages, with particular emphasis on the egg surface microbiota and their origin. We hypothesized that the egg surface microbiota is colonized either before oviposition, during oviposition, or actively after oviposition via inoculation by adult flies. Expected outcomes included identifying distinct bacterial communities at various life stages and sampling sites of eggs, with potential discovery of vertically transmitted microbes. To test this, we employed ten sampling approaches for the microbiota, from larval haemolymph and larval guts fed with sterilized and non-sterilized feed, pupal cell pulp, wash of ovipositor and eggs directly collected after oviposition, eggs dissected from the ovary and empty female abdomina, eggs of the fly cage with contact to adult BSF, and eggs of the fly cage with subsequent sterilization. Additionally, we screened the egg cytoplasmic microbiota, investigating the possible presence of microorganisms within. A feeding trial was conducted to evaluate the viability of neonate larvae after performing all sampling methods and to assess a difference in growth between larvae growing on sterilized feed and ones growing on non-sterilized feed. The microbiota were identified through 16 S rRNA gene sequencing to assess the stage in BSF development when the microbial colonization of the egg surface occurs.

## Methods

###  Breeding of Black Soldier Flies

A BSF colony in its 7th generation, originating from Hermetia Baruth GmbH (Baruth/Mark, Germany), was reared in an ICH750eco climate chamber (Memmert, Schwabach, Germany) at a temperature of 27 °C (± 0.5 °C) and 60% (± 0.5%) relative humidity as previously described [10, 38]. In brief, BSFL were kept in black polypropylene boxes (180 × 120 × 80 mm) sealed with plastic lids with integrated nets (a = 615 cm²) to allow aeration. Larvae were fed twice a week *ad libitum* with a 40:60 (w/v) mixture of ground chicken feed (Grünes LegeKorn Premium, Landwirtschaftliche Genossenschaft, Klagenfurt, Austria) and tap water. Pupae were transferred to white plastic cups (50 ml) and covered with wood shavings (Dehner Terra, Rain, Germany). Emerged adults were collected manually into transparent polypropylene cages (390 × 280 × 280 mm) with nets (fibreglass; 200 × 300 mm, mesh size 2 × 2 mm) integrated into the lids for aeration. The fly cages were illuminated with LED panels (Y51515227 184210, Barthelme, Nuremberg, Germany) in a 16:8 (L: D) photoperiod [39]. On two opposite wall cages, egg traps (corrugated black polypropylene cardboard) were installed for oviposition using magnets. A glass tube with tap water and plugged with cellulose paper was provided as a water source. Eggs were collected from the egg traps on Day 4 after the adults were introduced and were then placed in the above-mentioned larval boxes to hatch.

### Experimental Setup and Sampling

**Fig. 1:**
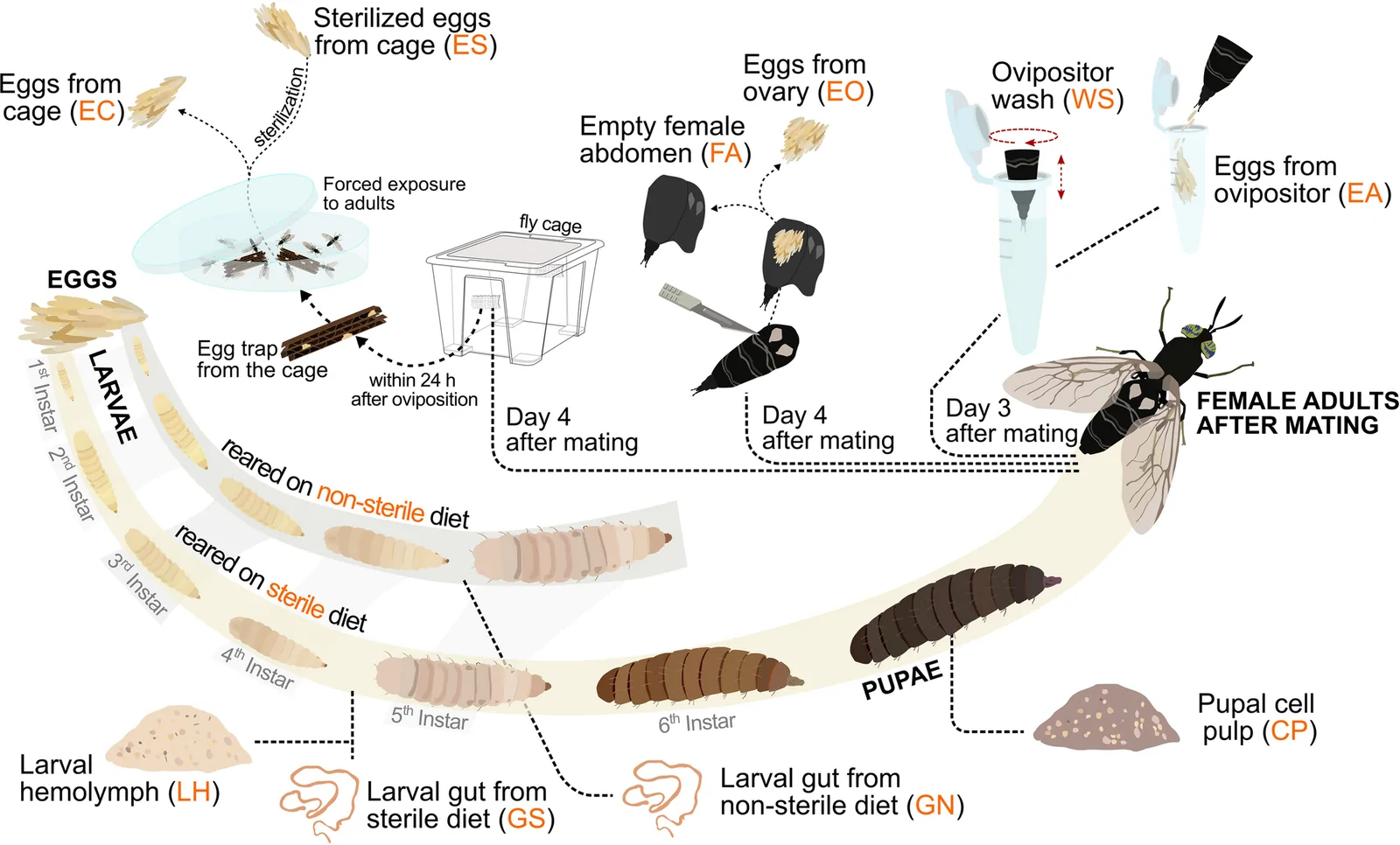
Illustration of the sampling procedure to assess egg surface microbiome of black soldier fly and its origin. Information on sampling procedures is outlined in Table 1 and detailed in *Online resource 1*

All BSF life stages used in this study were kept under the same conditions as mentioned in the previous section (“*Breeding of black soldier flies”*). Growth boxes were prepared by placing an egg trap containing ten egg clutches above freshly mixed chicken feed, with the trap secured in position using toothpicks. Before larval hatching, a thin layer of dry, ground chicken feed was spread along the inner edges of the boxes, as described in Addeo et al. (2022) [40], to prevent larvae from escaping. To avoid the introduction of exogenous microorganisms, ground chicken feed mixed with distilled water (40:60 w/v) and autoclaved before use. Larvae were fed *ad libitum* every other day, receiving feed amounts that substantially exceeded the known nutritional requirements based on both experience and literature [41], ensuring no limitation in feeding. Ten distinct sampling approaches (Fig. 1., Table 1; Online Resource (OR) 1) were applied to determine when, where, and which bacterial communities occurred during BSF development, and to identify the sources of microbial colonization. In addition to these treatments, a control group of larvae was raised on non-sterilized chicken feed to represent a non-axenic rearing environment. Samples for DNA extraction were collected in biological triplicates (n = 3); for each replicate, five individuals were pooled, except for the WS samples, where ten individuals were combined to obtain sufficient DNA. Further details on sample handling and extraction procedures are provided in the section “*DNA extraction*”.

**Table 1:** Overview of the ten different types of samples from which DNA was extracted for 16 S rRNA gene amplicon sequencing. A detailed description of sampling procedures is provided in *Online resource 1*

ID	Description	Sampling approach
GS	Larval gut from sterile diet	The whole gut was extracted from five surface sterilized larvae (50:50 mix (v/v) of 5% bleach and Milli-Q, followed by Milli-Q rinse [42, 43]) per replicate by pulling out the anus using sterile forceps and transferring it to sterile microcentrifuge tubes (> 0.05 g larval gut/replicate; *n* = 3).
GN	Larval gut from non-sterile diet	
LH	Larval haemolymph	For each replicate, the haemolymph from five larvae remaining after gut extraction was transferred to sterile microcentrifuge tubes (> 0.10 g larval haemolymph/replicate; *n* = 3).
CP	Pupal cell pulp	Five surface sterilized pupae (50:50 mix (v/v) of 5% bleach and Milli-Q, followed by Milli-Q rinse) per replicate were cut open along both lateral sides with sterile scissors, and the cell pulp was scraped out with a sterile spatula before transferring it to a sterile microcentrifuge tube (> 0.10 g pupal cell pulp/replicate; *n* = 3).
EC*	Non-sterilized eggs from cage	Egg traps containing eggs were collected from the cages and placed in a Petri dish together with 5 male and 5 female flies for 1 h to ensure contact between adults and eggs. Thereafter, 5 egg clutches per replicate were collected into sterile microcentrifuge tubes (> 0.05 g eggs/replicate; *n* = 3).
ES*	Sterilized eggs from cage	Five egg clutches per replicate (> 0.05 g eggs/replicate; *n* = 3) were directly collected from the cage’s egg traps and sterilized by first vortexing for 10 s and then incubating them for 2 min in 700 µl 50:50 mix (v/v) of 5% bleach and Milli-Q. The tubes were centrifuged (30 s at 11,000 × g), and the supernatant was removed. The pellet was washed following these steps: 700 µl of Milli-Q water was added; vortexed for 10 s; centrifuged (1 min/11,000 × g), the supernatant was removed, and these steps were repeated at least five times until the smell of bleach was no longer noticeable but before the egg surface started to break.
WS	Ovipositor wash	Ten gravid females per replicate were held above a sterile microcentrifuge tube filled with 700 µl lysis buffer SL1 (NucleoSpin Soil kit, Macherey-Nagel, Düren, Germany) one by one, and the ovipositor was dipped into the liquid and moved in circles for 1 min to wash microbes off of the ovipositor’s surface (*n* = 3).
EA*	Eggs from ovipositor	After washing the ovipositor, ten gravid females per replicate were decapitated to induce oviposition and eggs were directly collected in a sterile microcentrifuge tube (> 0.05 g eggs/replicate; *n* = 3).
EO*	Eggs dissected from ovary	Five gravid females per replicate were surface sterilized (50:50 mix (v/v) of 5% bleach and Milli-Q, followed by Milli-Q rinse) and cut open along the lateral sides of the abdomen to access and extract the ovary. The ovaries were transferred to a sterile microcentrifuge tube using a sterile spatula (> 0.05 g/replicate; *n* = 3).
FA	Empty female abdomen	After ovary extraction, the abdomina were separated from the thoraces and collected in a sterile microcentrifuge tube (> 0.05 g/replicate; *n* = 3).

*Viability of sampled eggs: To assess egg viability, three egg clutches in addition to the ones used for DNA extraction were collected for each of the sampling methods. The egg clutches were placed in separate sterile Eppendorf tubes positioned above non-sterilized feed within rearing boxes for larvae (n = 3), as described in the methods section “Breeding of black soldier flies”. The eggs were incubated under the same conditions as the rest of the animals (27 ± 0.5 °C and 60 ± 0.5% relative humidity) and monitored daily for hatching larvae. Larval growth was documented until first larvae started to transition to prepupal stage.

## Differences in Larval Growth on Sterilized and Non-Sterilized Feed

To assess whether there are differences in growth for larvae reared on sterilized and non-sterilized feed, we performed a feeding trial. BSFL were reared in triplicates (90 larvae per replicate; *n* = 3) on sterilized and non-sterilized chicken feed 40:60 (w/v) in 90 × 90 × 40 mm boxes. The BSFL were fed a ration of 100 mg/larva/day (fresh weight) [41]. Every other day, three times five larvae were randomly selected from each box (without replacement) and weighed to track their biomass gain. Growth was monitored until the first larvae began to transition to the prepupal stage, which in our laboratory colony typically occurred after approx. 14 days when reared on a chicken feed diet.

## Extraction of the Egg Cytoplasm and Isolation of Microorganisms

The extraction of egg cytoplasm was performed using a micromanipulator (M-152, Narishige) equipped with a capillary (BF100-78-10, Sutter Instrument) and connected to an inverted microscope (CKX53, Olympus). We extracted the egg cytoplasm from two different groups, each in three replicates: one consisting of a single individual and the other of five individuals. Immediately after extraction, the cytoplasm was immersed in 25 µL of Milli-Q water and then plated on Standard I nutrient agar (15 g L^− 1^ peptone, 3 g L^− 1^ yeast extract, 6 g L^− 1^ NaCl, 1 g L^− 1^ glucose, 12 g L^− 1^ agar; pH adjusted to 7.5). The plates were incubated at 27 °C for up to 72 h. Single colonies were picked using a heat-sterilized loop and transferred onto fresh agar plates via dilution plating.

## Colony PCR and Sanger Sequencing

A master mix consisting of 12.5 µL Taq 2X Master Mix with 1.5 mM MgCl2 (VWR, Radnor, PA, USA), 0.5 µL 27f primer (Eurofins Genomics, Ebersberg, Germany), 0.5 µL 1492r primer (Eurofins Genomics, Ebersberg, Germany), 0.5 µL 2% BSA (Thermo Fisher Scientific, Waltham, MA, USA) and 11 µL PCR-grade water (Carl Roth, Karlsruhe, Germany) per reaction was prepared for colony PCR. Following the aliquoting of the master mix into 0.2 mL reaction tubes, bacterial isolates were carefully picked by gently scratching the colonies using sterilized pipette tips. The tip of the pipette tip was directly submerged in the PCR reaction mix and gently stirred to detach the harvested bacterial biomass (PCR protocol in OR1). PCR products were purified using a GenElute PCR Clean-Up Kit (Sigma-Aldrich, St. Louis, MO, USA) and eluted using the enclosed elution buffer. Purified DNA was quantified via UV-Vis spectrophotometry (NanoDrop 2000c, Thermo Fisher Scientific, Waltham, MA, USA), and DNA quality was assessed via gel electrophoresis. DNA passing the quality control was sent to Eurofins Genomics (Ebersberg, Germany) for Sanger sequencing using the 27f primer. The returned sequences were aligned to the 16 S ribosomal RNA sequences database via nucleotide BLAST^®^ search and using the megablast tool for highly similar sequences.

### DNA Extraction and 16 S rRNA Gene Amplicon Sequencing

DNA from the ten types of samples (Table 1) was extracted using the NucleoSpin Soil kit (Macherey-Nagel, Düren, Germany) following the manufacturer’s protocol with some modifications: the lysed sample of the larval haemolymph was vortexed for 10 min, and the other samples for 5 min. Prior to precipitation with SL3 Buffer, the supernatant was moved to a new collection tube. DNA was eluted twice using 20 µl SE Elution Buffer each. DNA concentration and quality were checked via gel-electrophoresis and UV-Vis spectrophotometry (NanoDrop 2000c, Thermo Fisher Scientific, Waltham, MA, USA), and DNA was stored at −20 °C until further processing.

An enrichment PCR of all samples was performed by the sequencing provider due to low DNA concentrations in some samples measured via UV-Vis spectrophotometry. The enrichment was performed by diluting (2.5 ng/µl) the samples, followed by an enrichment PCR with locus-specific primers (V34:IlluminaF TCGTCGGCAGCGTCAGATGTGTATAAGAGACAGNNNNN*CCTACGGGNGGCWGCAG*; IlluminaR GTCTCGTGGGCTCGGAGATGTGTATAAGAGACAGNNNNN*GACTACHVGGGTATCTAATCC*, *italics* = locus-specific sequences). Then, a 1-step PCR with locus-specific primer and Illumina overhang and a cleanbead purification were performed, followed by a 2-step PCR with index primer and another cleanbead purification. The final libraries were pooled, and a final cleanbead purification of the pool was carried out. Illumina MiSeq amplicon 16 S genetic sequencing was performed by Microsynth AG (Balgach, Switzerland) using a 2 × 250 bp paired-end approach with the universal bacterial primers 341f (5′-CCTACGGGRSGCAGCAG-3′) and 802r (5′-TACNVGGGTATCTAATCC-3′) targeting the V3-4 region on the 16 S rRNA gene. Library preparation was performed by the sequencing provider based on a Nextera two-step PCR including purification, quantification, and equimolar pooling. In addition, the ITS2 genetic region was sequenced using the ITS3f (5′-GCATCGATGAAGAACGCAGC-3′) and ITS4r (5′-TCCTCCGCTTATTGATATGC-3′) primer pair. However, due to the low quality of the reads resulting from this sequencing job, we decided to exclude the data on fungal communities from further analyses and interpretation.

## Processing and Analysis of Sequencing Data

Raw reads generated by targeting the V3-V4 genetic region were filtered, trimmed, and dereplicated in DADA2 v1.8 following the standard operating procedure [44]. After inferring amplicon sequence variants (ASVs), the paired forward and reverse reads were merged, and chimeras were removed. Taxonomy was assigned using the reference databases SILVA (v.132; Quast, et al. (2012) [45]). The data were visualized using ampvis2 (v.2.7.4; Andersen, et al. (2018) [46]) and ggplot2 (v.3.3.5; Wickham (2016) [47]). Venn diagrams were created using the MicEco package (v.0.9.17; Russel (2022) [48]). The network was calculated from sample-wise distances based on Bray-Curtis dissimilarity using the make_network() command with max.dist = 0.5 and visualized using the plot_network() command from the phyloseq package (v. 1.46.0 [49]),.

Reproducible documentation of sequence processing and data analysis as well as download options for relevant data can be accessed via https://tklammsteiner.github.io/stagespecificmicrobiota.

### Statistical Analysis

Alpha diversity (Shannon index) and linear discriminant analysis of effect size (LEfSe; threshold at LDA score (log10) > = 2) were calculated using the microbiome (v.1.16.0; Lahti, et al. (2017) [50] and microbiomeMaker (v. 1.0.1; Yang (2021) [51] package, respectively. Differences between means of alpha diversity indices were calculated via Wilcoxon test. Permutational analysis of variance (PERMANOVA) was calculated based on Bray-Curtis dissimilarity values using the adonis function (permutations = 1000) in vegan (v.2.5–7.5; Oksanen, et al. (2020) [52]). Pairwise differences in microbial community composition of treatment groups were assessed using the pairwise.perm.manova function (nperm = 1000) with subsequent Bonferroni correction in RVAideMemoire package (v.0.9.81; Hervé (2022) [53]). The results were considered statistically significant when their p-value was < 0.05.

## Results

### An Extensive Transition in Family-Level Relative Abundance during BSF Development

**Fig. 2:**
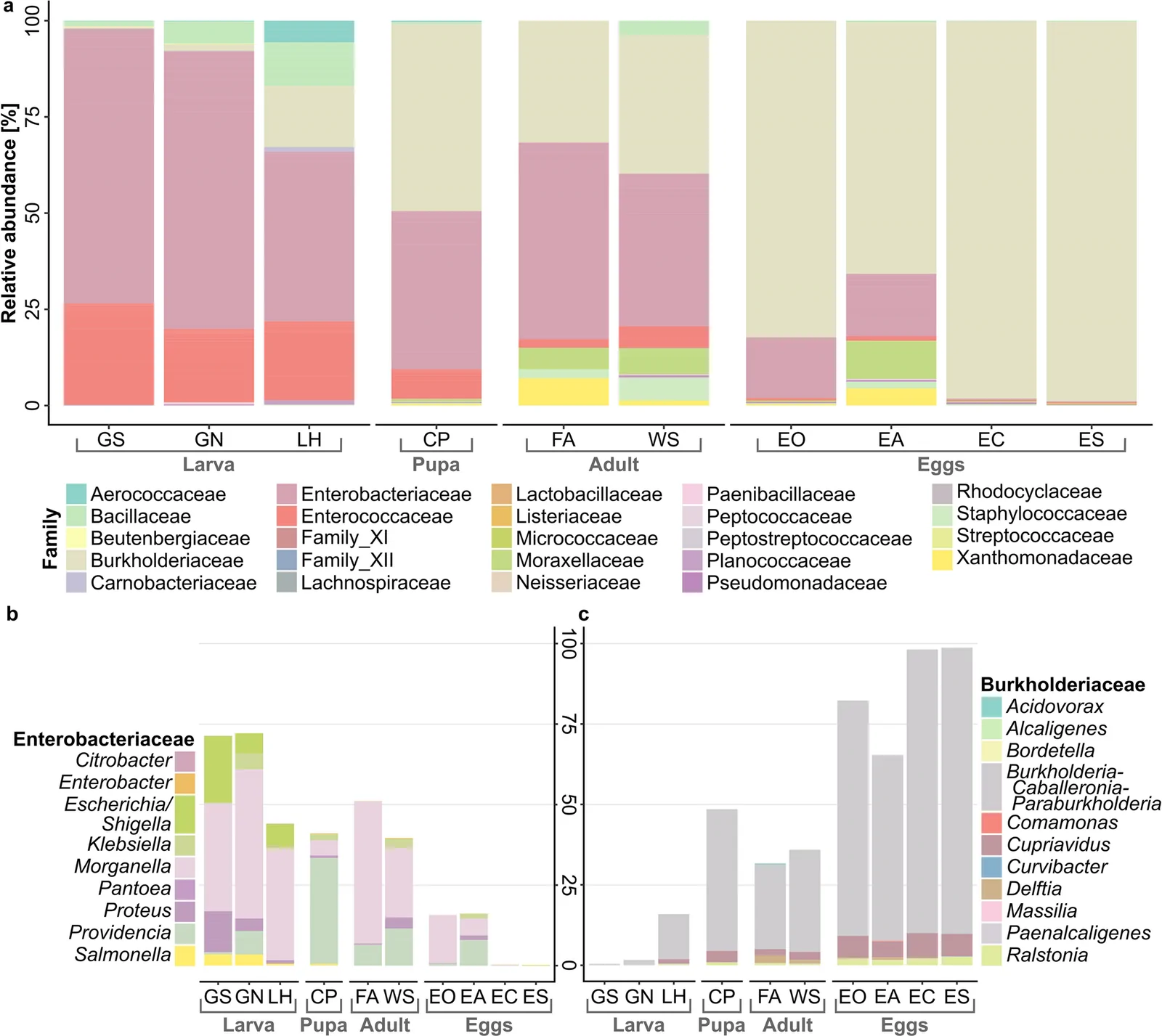
**(a)** Community composition at family level. Genus-level composition of the families **(b)** Enterobacteriaceae and **(c)** Burkholderiaceae in different life stages of the black soldier fly: Larvae fed with non-sterile (GN) and sterile (GS) feed and the larval haemolymph of GS (LH), the pupal cell pulp (CP), and from the female adults after mating, a wash of the ovipositor (WS) and the afterwards placed eggs of the ovipositor (EA), eggs collected from the ovary (EO) and the empty female abdomen (FA), eggs collected from a fly cage after forced exposure to adults (EC) and sterilized (ES)

An average of 31,698 ± 11,232 raw reads per library were generated by Illumina MiSeq amplicon sequencing. After filtering, denoising, and chimera removal, 26,125 ± 8,914 high-quality reads remained, which were further rarefied to the smallest sample size (16,167 reads) before subsequent biostatistical analysis. Due to an inadequately low read number (679 reads), sample EA1 (replicate 1 of eggs from ovipositor) was removed as an outlier in the process of subsampling. At the family level, the highest relative abundance for bacteria in all larval stages was Enterobacteriaceae, though less dominant for LH, followed by Enterococcaceae (Fig. 2a, OR2: *Fig. **S1*). In LH, the families Aerococcaceae, Bacillaceae, and Burkholderiaceae were also highly abundant. In terms of genus-level representatives of Enterobacteriaceae, *Morganella.* was most abundant for all larval stages (GN, GS, and LH), followed by *Escherichia* and *Proteus* (Fig. 2b). The abundance of Enterobacteriaceae decreased during the prepupal stage and was similarly abundant as Burkholderiaceae in the CP samples. Within the family of Enterobacteriaceae, *Providencia* was the most abundant genus in CP. This trend shifted slightly in the adult stage, in which the abundance of Enterobacteriaceae increased again, but Burkholderiaceae were still highly present. In all egg samples, Burkholderiaceae accounted for most of the classified sequences, with *Burkholderia-Caballeronia-Paraburkholderia* as the most abundant genus-group dominating EC and ES samples. Similarities were found in microbiota composition between the FA, WS, and EA samples, showing the presence of Xanthomonadaceae, Micrococcaceae, and Staphylococcaceae.

### Diversity in the Egg Surface Microbiome during BSF Development and Unique and Shared ASVs

**Fig. 3:**
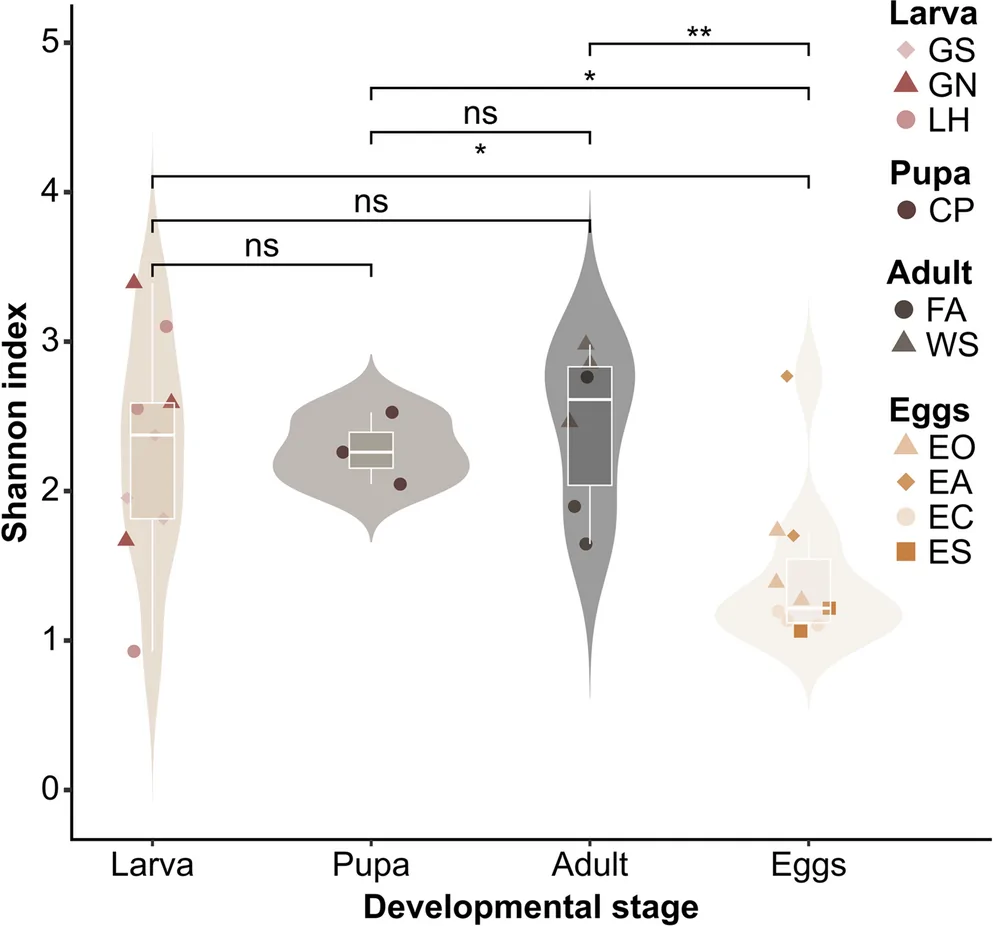
Shannon diversity index (NS: *p* = 1, ns: *p* > 0.05, *: p < = 0.05; **: p < = 0.01) for the microbial communities of various black soldier fly life stages: Larvae fed with non-sterile (GN) and sterile (GS) feed and the larval haemolymph of GS (LH), the pupal cell pulp (CP), and from the female adults after mating, a wash of the ovipositor (WS) and the afterwards placed eggs of the ovipositor (EA), eggs collected from the ovary (EO) and the empty female abdomen (FA), eggs collected from a fly cage after forced exposure to adults (EC) and sterilized (ES)

As expressed by the Shannon diversity index (Fig. 3), the egg stage had a significantly lower diversity compared with the larval (p = 0.020) and pupal stages (p = 0.038) as well as the adult stage (p = 0.004). In the larval stage, GN had the highest diversity (H’ = 2.6 ± 0.9) and LH the lowest (H’ = 2.2 ± 1.1). For the adult stage, WS had a higher diversity (H’ = 2.8 ± 0.3). For the egg stage, the highest diversity was observed in the EA samples (H’ = 2.2 ± 0.8), while all of the ES samples had a relatively low diversity (H’ = 1.1 ± 0.1).

**Fig. 4:**
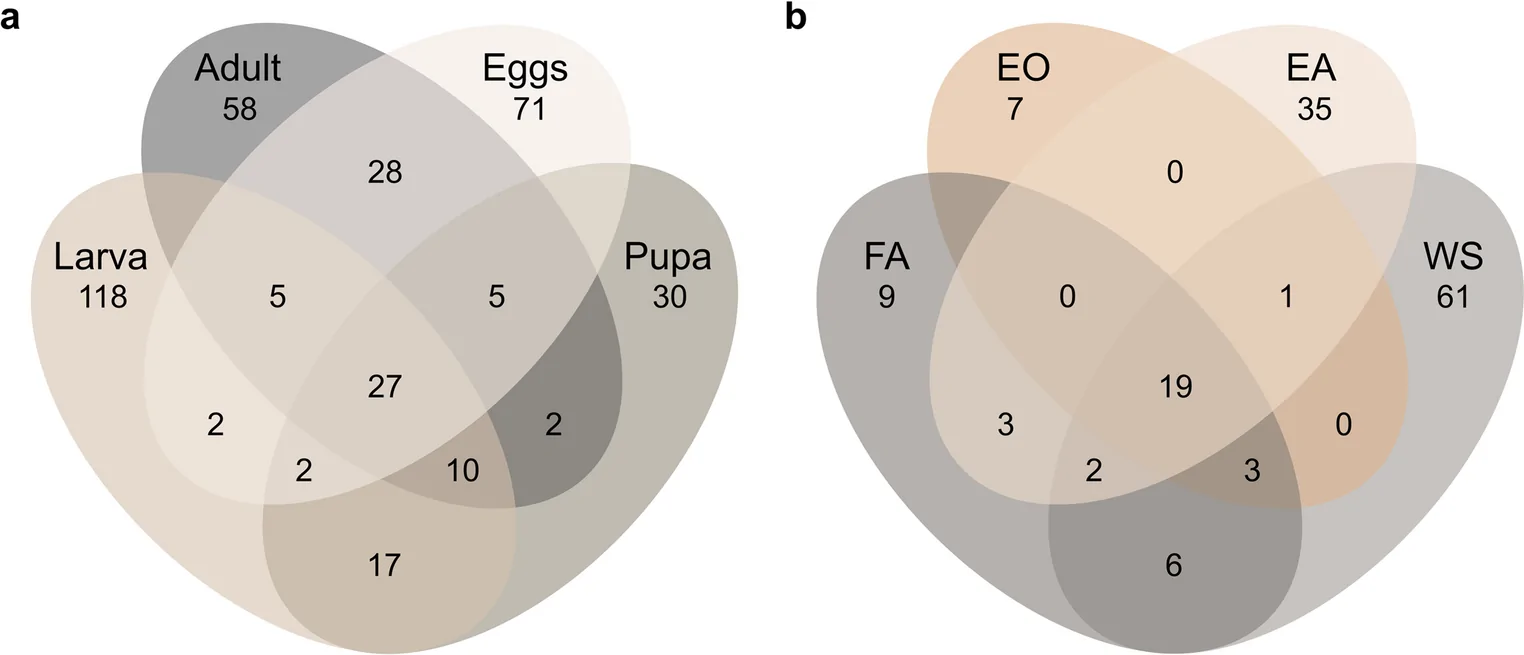
Venn diagram showing unique and shared ASVs for (**a**) different life stages of black soldier fly and (**b**) comparison between adult and egg stages collected from the female adults after mating, a wash of the ovipositor (WS) and the afterwards placed eggs of the ovipositor (EA), eggs collected from the ovary (EO) and the empty female abdomen (FA)

The highest number of unique ASVs (ASVs not shared with other life stages) was found in the larval stage (118 ASVs, Fig. 4a), while the lowest number of unique ASVs was found in the pupal stage (30 ASVs). The most ASVs shared exclusively between two life stages were found between the adult and egg stages (28 ASVs) and the lowest shared ASVs were between larva and egg, and pupa and adult (2 ASVs). Among the sampling approaches WS, EA, FA, and EO (Fig. 4b), the highest unique ASVs for adults were found via WS (61 ASVs), whilst via FA only 9 unique ASVs were found. Originating from the same treatment, the highest number of unique ASVs for the egg stage was found in the EA (35 ASVs), and similarly low to FA was the number of unique ASVs found in EO (7 ASVs). The highest number of ASVs shared exclusively between two groups was found between WS and EA (22 ASVs), while FA and EO only shared 3 ASVs. Among EO and WS, and FA and EO, the exclusively shared ASVs were 0. However, WS and FA shared 6 ASVs.

**Fig. 5:**
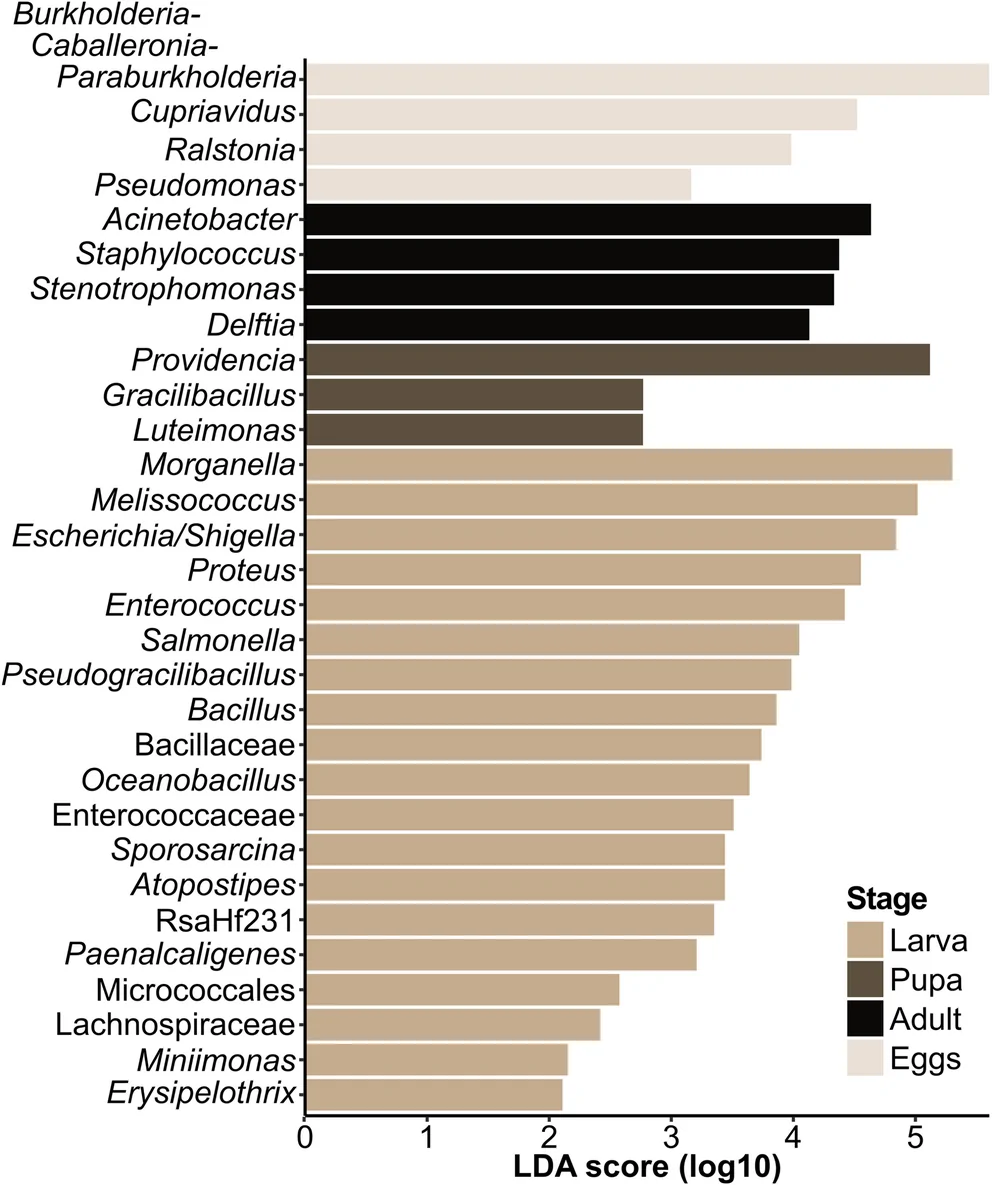
Linear discriminant analysis of effect size (LEfSe) identified characteristic bacterial genera (or families, where genus was not reliably identified) for each developmental stage. Samples were grouped based on their life stage, whereas the “Larva” group consisted of samples obtained from larvae fed with non-sterile diet (GN), larvae fed with sterile diet (GS), and haemolymph from larvae fed with sterile diet (LH) samples; The “Pupa” group consisted of samples from pupal cell pulp (CP), the “Adult” group consisted of samples from the empty female abdomen (FA) and ovipositor wash (WS); The “Eggs” group consisted of samples from eggs collected from the ovary (EO), eggs deposited by the ovipositor (EA), eggs collected from a fly cage after forced exposure to adults (EC), and surface-sterilized eggs (ES). The threshold for biomarker identification was set to LDA score (log10) > = 2

Biomarker analysis based on LEfSe further distinguished significantly overrepresented bacterial genera for larval, pupal, adult, and egg developmental stages (Fig. 5). The *Burkholderia-Caballeronia-Paraburkholderia* genus was found to be characteristic for egg samples, while the genera *Acinetobacter*, *Staphylococcus*, and *Stenotrophomonas* were similarly overabundant in adult samples. *Providencia*, which had high relative abundances also in adults and eggs, was by far the most overrepresented genus in pupal samples. Among the investigated BSF life stages, the complexity of the larval gut microbiota put forth the most biomarker taxa passing the threshold of LDA > = 2, with *Morganella*, *Melissococcus*, and *Escherichia/Shigella* scoring highest.

PERMANOVA based on Bray-Curtis distances confirmed that there were significant differences in microbial communities across stages (*p* = 0.001; larvae, pupae, adult, egg), as well as across tissue samples (*p* = 0.001; larval guts, larval haemolymph, adult haemolymph, ovipositor, ovarium, and eggs) (OR 3: *Tab. S1*). Pairwise significant differences in microbial community composition were found across all developmental stages, except between pupae and adults, and across all tissue samples, except between tissue of ovarium and tissue of haemolymph and ovipositor (OR 3: *Tab. S2*).

### Viability of Eggs and Larval Growth

Hatching larvae were observed only for the EA and EC sampling procedures and show successful growth (OR2: *Fig. **S3** and S4*). The eggs from the EO and ES sampling procedures were not viable. No significant differences were found in the growth of larvae fed with sterilized and non-sterilized feed (OR2: *Fig. S4*).

### Bacterial Isolates from the Egg Cytoplasm

Five bacterial isolates were obtained from the microbiological cultivation of wash solutions derived from BSF eggs. The isolates were grouped into two distinct morphotypes based on colony morphology. Bacterial growth occurred only on two culture plates that contained cytoplasmic material extracted from a single egg. Sanger sequencing of all isolates confirmed the morphological grouping and identified two taxa: *Bacillus zanthoxyli* (Isolates 1 and 2) and *Dermacoccus nishinomiyaensis* (Isolates 3, 4, and 5) (OR3: *Tab. S3*).

## Discussion

This study analyzed the microbiota of different BSF life stages, with a special focus on the egg surface microbiota, and to determine at which developmental stage and how the microbial colonization of the eggs occurs. Our results indicate that a gradual transition in bacterial community structure occurs during BSF development, from an Enterobacteriaceae-dominated community in larval stages to a Burkholderiaceae-dominated community in egg stages. Furthermore, the results indicate that inoculation of the dominant bacterial community on the egg surface occurs before oviposition, as the relative abundance in the EO samples shows.

The composition of bacterial communities in the gut of the GN and GS larvae was similar, indicating little to no influence on sterilization of the feed. In contrast, Schreven et al. [30] found that sterilization of chicken manure prior to feeding it to BSFL led to significant changes in microbiota composition and negatively affected larval growth. Sterilization of the egg surface had no notable consequences, indicating that substrate-borne microorganisms may be more important to larval development. In studies using BSFL fed with non-sterilized chicken feed, *Gammaproteobacteria* (Enterobacteriaceae, *Morganella*) and *Bacilli* (*Enterococcus*, *Lactococcus*) were the most abundant phyla, thus supporting our results (Fig. 2a and b) [19, 38, 54, 55]. Genera such as *Morganella*, *Enterococcus*, and *Providencia* were significantly overrepresented in larval and pupal samples and have previously been found to play a key role in the BSFL core gut microbiome (Fig. 5; [38]). In LH, the relative abundance of Enterobacteriaceae decreased, though still dominant, while Bacilli and Burkholderiaceae increased. Representatives of the Bacilli class have often been associated with BSF larvae. They promote their growth by fermenting organic matter and, thus, facilitating its uptake and digestion [38, 56, 57]. Burkholderiaceae have been rarely detected, and their role in the BSF life cycle is unknown. In a study performed by Zheng, et al. (2013) [33], Burkholderiaceae were identified throughout all life stages of BSF, though classified as a minor component of the fly’s microbiome. In the CP samples, Enterobacteriaceae were almost as abundant as Burkholderiaceae. This trend shifted in the adult stage, during which Enterobacteriaceae made up the major share of reads, though Burkholderiaceae were still heavily represented. In addition, Xanthomonadaceae, Micrococcaceae, and Staphylococcaceae were increasingly detected in the adult stage. These families have previously been associated with BSFL [18, 33, 38]. In other insect species such as the pumpkin fruit fly (*Zeugodacus tau*), the same bacterial families have been reported to possibly serve as a source of vitamins, nitrogen, and amino acids, and some of them can be transmitted vertically from the parents to their offspring [58]. A similar population structure of bacteria was found in the EA treatment, indicating that an inoculation through the ovipositor occurs, as this treatment consisted of freshly oviposited eggs. Furthermore, the EO samples showed a heavy dominance of Burkholderiaceae and a strong presence of Enterobacteriaceae, but unlike EA, no other bacterial groups were highly represented, indicating an inoculation at the time of oviposition. Interestingly, most other bacterial groups on the older eggs (EC and ES) were outcompeted by Burkholderiaceae. Some members of the Burkholderiaceae can function as opportunistic pathogens, and some have the capacity to degrade chlororganic pesticides [33]. A study has shown that a stinkbug may harbour some fenitrothion-resistant *Burkholderia* from the environment, and these bacteria can be an easy way for the insect to detoxify insecticides [59]. *Burkholderia-Caballeronia-Paraburkholderia* were the most dominant genera found among the Burkholderiaceae throughout all life stages and were identified as characteristic colonizers of BSF eggs (Figs. 2c and 5). These taxa can interact with their host by supplementing specifically required forms of nitrogen and other nutrients to ensure normal development of, for example, xylophagous insects [60].

Studies showed that the evolutionary success of insects is widely attributed to their diverse relationships with beneficial microorganisms [61, 62]. The features of bacterial communities across the life stages of BSF indicate that microbes are tightly linked to the fly’s development and growth, which includes maturation of the immune system, resistance to chemical substances (e.g., insecticides), supplementation of nutrients, and digestion. Microbiological surveys of BSF populations across habitats harbour a highly diverse yet relatively consistent internal bacterial community, regardless of geographic location, while diet can influence the bacterial communities [38, 61, 63]. The manipulation of the microbiota in mass-reared insects is proposed to enhance insect rearing by utilizing and strengthening microbiota-related strategies [37]. This manipulation could focus on utilizing host-associated beneficial microbes to fortify health and immunity [61]. However, for this strategy to be effective in the mass rearing of BSF, more detailed information on the impact of the microbiome and of specific microbiota on the immune functioning of these insects is needed [61].

Similarly, the Shannon diversity index indicates that bacterial community composition experiences changes in community complexity during the BSF life cycle (Fig. 3). The bacterial diversity was higher during larval and adult stages and decreased for the pupal stage and reached a significantly low level for egg stages. This is not surprising, considering BSF eggs are immobile and pupae are semi-immobile and do not feed anymore and, therefore, have less exposure to environmental and transient microbes [33]. The lowest diversity was observed for the ES treatment indicating that sterilisation of the egg surface can be considered effective. However, to assess if trans-generational immune priming occurs via bacterial injection into the developing egg, further analyses testing the sterilization should be performed [56]. Other authors [64] reported that surface sterilization of eggs led to a drastic reduction of hatch rates, making only approx. 30% of eggs viable, while inoculation of eggs with *Bacillus subtilis* and *Staphylococcus saprophyticus* improved hatching in both untreated and sterile eggs. Zheng, et al. (2013) [18] showed that sterilized eggs lead to a lower percentage of oviposition. This implies that the bacteria present in non-sterilized eggs may attract conspecific gravid females, a phenomenon also observed in other dipterans such as mosquitoes [65].

A subset of 27 bacterial ASVs was present in all life stages (Fig. 4a), whereas 28 ASVs were exclusively shared between adults and eggs and an additional 17 ASVs between larvae and pupae. This similarity in taxonomic features indicates the transmission of bacteria across life stages. The larval and egg stages had 118 and 71 unique ASVs, respectively, that were not present in other life stages, indicating a distinct community structure. To assess if and to what degree an inoculation of eggs by adult females might take place, constellations of unique and shared ASVs between FA, EO, WS, and EA samples were visualized in a Venn diagram (Fig. 4b). These samples consist of eggs sampled directly from the female abdomen and the emptied female abdomen, a wash of the ovipositor and the eggs that were directly oviposited afterwards. From this analysis, a profile of shared bacterial groups associated with females and eggs post- and pre-oviposition was derived. The results show that with 22 bacterial ASVs exclusively shared between EA and WS, it is likely that an inoculation occurs during the oviposition process. Furthermore, the EA has no exclusively shared features with the EO eggs, except for the 19 ASVs present in all four treatments, indicating no similarities before and after oviposition. However, the FA samples shared only three ASVs with EA, except for the 19 ASVs present in all four treatments, indicating that the bacterial community of adult females is not similar to that of freshly oviposited eggs before oviposition starts. This could mean that females accumulate certain bacteria during oviposition to inoculate eggs with a specific community. A similar progression can be derived from the diagrams of community compositions (Fig. 2).

The larvae fed with non-sterilized and sterilized feed did not differ in their growth performance, although higher mortality was observed for the non-sterilized feed, while the sterilized feed resulted in a lower pupation rate (Fig. S4). Notably, the sampling method EA yielded viable larvae (OR2: *Fig. **S3*), which is particularly intriguing for laboratory approaches requiring eggs without contact with external substances.

In the context of this study, we analysed the egg cytoplasm of BSF to explore the potential presence of intraovular microbial communities. The extracted cytoplasm was cultivated on standard nutrient agar with the intention of providing a broad nutrient source for the growth of aerobic microorganisms, while recognizing that this approach might exclude the growth of bacteria requiring more specific environmental conditions and nutrient supply. Notably, we identified *Bacillus zanthoxyli* in a single egg sample, a finding previously unreported in BSF. *Dermacoccus nishinomiyaensis* has been observed in the larval guts in a study performed by Klüber et al. (2022) [66], hinting at its potential association with the BSF life cycle. Nonetheless, we must exercise caution in conclusively suggesting the presence of *Bacillus* and *Dermacoccus* inside the BSF eggs, as neither of these species was consistently detected in any of the treatments analysed in depth. Especially, despite implementing a bleach treatment to eliminate cuticular bacterial DNA, the detection of the major bacterial DNA associated with BSF eggs yielded results similar to those reported by Binetruy et al. [67], which focused on the removal of external bacterial DNA to analyse internal bacterial DNA of ticks. These findings highlight the complexities in accurately characterizing the egg cytoplasm microbiota of BSF and emphasize the need for more comprehensive studies to analyse its true composition and significance during BSF development.

This study analysed the microbiota on the egg surface and their origin and advances our understanding of the BSF microbiota by illuminating the dynamic changes in microbial community composition throughout ontogeny, particularly highlighting the dominant and persistent presence of Burkholderiaceae in the egg stage. The presence of this family of bacteria through all life stages suggests a potential transmission mechanism, which becomes especially noticeable between the adult and egg stages during oviposition (EA and WS, Fig. 4b). By demonstrating that Burkholderiaceae outcompete other taxa post-ovipostion independent of active inoculation by adult flies (Figs. 2a and c and 3), this study challenges existing speculations about the acquisition of microbiota in BSF and opens up new research trajectories to explore pathways of microbial transmission and ecological interactions during insect development. These trajectories could involve studying eggs separated from adults immediately after oviposition to clarify means of transmission. Additionally, exploring the functional role of dominant egg-associated bacteria in BSF immunity, digestion, and detoxification processes through metagenomic and functional assays could illuminate how the microbiota contribute to larval performance and bioconversion efficiency. Future research could also benefit from comparing pupae and adult stages derived from larvae raised on both sterile and non-sterile diets to gain a more comprehensive understanding of how diet sterility influences insect development and physiology beyond the larval stage, adding depth to the assessment of microbial and nutritional impact in insect farming studies. Broader implications of our study extend to improving BSF rearing practices by enabling microbial monitoring and management for enhanced larval growth, pathogen resistance, and bioconversion.

## Conclusion

The characterization of bacteria associated with the developmental stages of BSF contributes to understanding the important role of microorganisms in interkingdom interaction. Our results indicate a high abundance of Burkholderiaceae in the ES treatment as well as in the EC treatment. Further investigations should be performed to analyse if Burkholderiaceae are mainly inside the egg or on its surface. Decrypting this interkingdom interaction could help to manipulate and optimize the process of oviposition via artificial inoculation of a substrate with microbial attractants. This might contribute to a future breakthrough for BSF industrial rearing, as larval loss caused by uncoordinated oviposition could be minimized. Furthermore, by analysing bacterial communities of BSF life stages, this study improves the understanding of the transfer of possible pathogens between life stages and generations.

## Supplementary Information

Below is the link to the electronic supplementary material.

Supplementary Material 1

Supplementary Material 2

Supplementary Material 3

## Data Availability

The datasets generated during the current study are available in the EMBL European Nucleotide Archive (ENA) repository under accession BioProject accession number PRJNA809118, https://www.ebi.ac.uk/ena/browser/view/PRJNA809118. Detailed documentation on sequence preprocessing, statistical analysis, and visualization is available under the following URL: https://tklammsteiner.github.io/stagespecificmicrobiota/.
