# Supplementary material for: Stage-Specific Microbiota Transitions Throughout Black Soldier Fly Ontogeny
Source: Microb Ecol. 2026 Jan 10;89:41. doi: 10.1007/s00248-025-02691-1 (PMC12860825; doi:10.1007/s00248-025-02691-1)
Supplement: Supplementary file 1 [file 248_2025_2691_MOESM1_ESM.docx]

**Stage-specific microbiota transitions throughout black soldier fly ontogeny**

Thomas Klammsteiner^1^, Carina D. Heussler^1^, Katharina T. Stonig^1^, Heribert Insam^2^, Birgit C. Schlick-Steiner^1^, Florian M. Steiner^1^

^1^ Universität Innsbruck, Department of Ecology, 6020 Innsbruck, Austria

^2^ BioTreaT GmbH, Technikerstr. 21d, 6020 Innsbruck, Austria

**Online Resource 1**

**Detailed sampling procedures**

*Collection of larval haemolymphs and guts from non-sterilized and sterilized diet*

Nine days after hatching (DAH), about 50 same-sized larvae (approx. 4 to 5^th^ instar) were collected and stored at -20 °C for DNA extraction and as backup, in estimation of half-life at 18 days until first pupation through self-observation and literature[37]. Frozen larvae were thawed for two min in a solution of 50:50 5% bleach (Danklorix, CP GABA, Hamburg, Germany) and Milli-Q (Merck, Darmstadt, Germany) water for surface sterilization [42, 43] and then placed in a 50-ml tube filled with pure Milli-Q water. The whole gut of five larvae was extracted by pulling out the anus using sterile forceps to assure complete extraction and transferred into a sterile microcentrifuge tube (resulting in at least 0.05 g/replicate; n = 3) [38]. The remaining haemolymph was placed in an empty and sterile microcentrifuge tube (at least 0.10 g/replicate; n = 3). The same procedure was used to collect the gut of the larvae fed with the non-sterilized diet (n = 3).

*Collection of pupal cell pulp*

About 50 pupae were collected 19 DAH and stored at -20 °C. Frozen pupae were thawed for two min in a solution of 50:50 5% bleach and Milli-Q water for surface sterilization, and afterwards, pupae were placed in a 50 ml tube filled with Milli-Q water. Each pupa was cut along both lateral sides with sterile scissors and the cell pulp was scraped out with a sterile spatula (five pupae per replicate). The cell pulp was collected into empty and sterile microcentrifuge tubes (at least 0.10 g/replicate; n = 3).

*Collection of wash of the ovipositor and eggs immediately after oviposition*

To assure easy handling and to prevent flies from escaping while collecting gravid females, the number of flies per cage was limited to a density of 0.0033 flies/cm^3^. Fifty females and 50 males aged 24 h were released into the fly cages. A total of three fly cages were used to obtain enough females for all sampling approaches, and the sampling of individuals occurred randomly across all cages. The fly cages were kept under the same conditions (see section “*Breeding of black soldier flies*”). To obtain enough gravid females it was assumed that females start oviposition on Day 4 after being transferred to the cages [39]. Accordingly, the collection of females for this treatment was set on Day 3 following the transfer of flies to the cages. Gravid females were collected manually one at a time. Each female was held above a sterile microcentrifuge tube filled with 700 µl lysis buffer SL1 (NucleoSpin Soil kit, Macherey-Nagel, Düren, Germany), and the ovipositor was dipped into the liquid and moved in circles for one min to wash microbes off of the ovipositor’s surface (WS) (n = 3). Thereafter, the female was decapitated to induce oviposition. Each female was held above a sterile microcentrifuge tube to allow oviposition into the tube (EA; at least 0.05 g/replicate; n = 3).

*Collection of the ovarian eggs and the empty female abdomen*

Approximately 20 gravid females were collected on Day 4 after transferring flies to the fly cages and stored at -20 °C. Frozen females were thawed for two min in a 50:50 solution of 5% bleach and Milli-Q water for surface sterilization and placed in a 50 ml tube filled with Milli-Q. To access the ovary, five females per replicate were cut along both lateral sides of the abdomen with sterile scissors, and the ovary was collected into a sterile microcentrifuge tube using a sterile spatula (at least 0.05 g/replicate; n = 3). The remaining abdomina of the females were separated from the thorax and collected into sterile microcentrifuge tubes (at least 0.05 g/replicate; n = 3).

*Collection of eggs from the fly cage after contact with adult BSF*

The remaining flies in the fly cages were allowed to oviposit. On Day 6 after transferring the flies into the fly cage, the flutes were collected into a sterile Petri dish. Five females and five males were introduced into the Petri dish and left for one h at 27 °C and 60% relative humidity. This procedure assured contact between the eggs and adults and enabled inoculation of eggs with adult-derived microbes. Thereafter, five egg clutches were collected into sterile microcentrifuge tubes (at least 0.05 g/replicate; n = 3).

*Collecting of sterilized eggs*

Five egg clutches were collected into sterile microcentrifuge tubes (at least 0.05 g/replicate; n = 3). The tubes were filled with 700 µl of a 50:50 mixture of 5% bleach and Milli-Q Water, vortexed for 10 s, and incubated for 2 min. The tubes were centrifuged (30 s at 11,000 × g), and the supernatant was removed. The pellet was washed following these steps: 700 µl of Milli-Q water was added; vortexed for 10 s; centrifuged (1 min /11,000 × g), the supernatant was removed, and these steps were repeated at least five times until the smell of bleach was no longer noticeable but before the egg surface started to break.

**PCR Protocol**

The following protocol was used for PCR amplification:

Step 1: initial denaturation at 95 °C, 5 min

Step 2: denaturation at 95 °C, 30 s

Step 3: annealing at 53 °C, 30 s

Step 4: elongation at 72 °C, 45 s

Step 5: final elongation at 72 °C, 10 min

Step 6: storage at 12 °C until termination

Steps 2-4 were cycled 30 times.

**References from the main text**

[37]	De Smet J, Wynants E, Cos P, Campenhout LV (2018) Microbial community dynamics during rearing of black soldier fly larvae (*Hermetia illucens*) and its impact on exploitation potential. Appl Environ Microbiol 84:e02722-17. https://doi.org/10.1128/AEM.02722-17

[38]	Klammsteiner T, Walter A, Bogataj T, et al (2020) The core gut microbiome of black soldier fly (*Hermetia illucens*) larvae raised on low-bioburden diets. Front Microbiol 11:993. https://doi.org/10.3389/fmicb.2020.00993

[39]	Heussler CD, Walter A, Oberkofler H, et al (2018) Influence of three artificial light sources on oviposition and half-life of the black soldier fly, *Hermetia illucens* (Diptera: Stratiomyidae): Improving small-scale indoor rearing. PLOS ONE 13:e0197896. https://doi.org/10.1371/journal.pone.0197896

[42]	Amiri E, Kryger P, Meixner MD, et al (2018) Quantitative patterns of vertical transmission of deformed wing virus in honey bees. PLoS ONE 13:e0195283. https://doi.org/10.1371/journal.pone.0195283

[43]	Hausmann A, Höcherl A, Niessner A, et al (2021) Accurate decontamination of insects from bulk samples does not affect DNA sequencing success. Spixiana 44:71–76
