# Supplementary material for: Stage-Specific Microbiota Transitions Throughout Black Soldier Fly Ontogeny
Source: Microb Ecol. 2026 Jan 10;89:41. doi: 10.1007/s00248-025-02691-1 (PMC12860825; doi:10.1007/s00248-025-02691-1)
Supplement: Supplementary file 2 [file 248_2025_2691_MOESM2_ESM.docx]

**Stage-specific microbiota transitions throughout black soldier fly ontogeny**

Thomas Klammsteiner^1^, Carina D. Heussler^1^, Katharina T. Stonig^1^, Heribert Insam^2^, Birgit C. Schlick-Steiner^1^, Florian M. Steiner^1^

^1^ Universität Innsbruck, Department of Ecology, 6020 Innsbruck, Austria

^2^ BioTreaT GmbH, Technikerstr. 21d, 6020 Innsbruck, Austria

**Online Resource 2**

**Fig. S1.** Heatmap of microbial communities at genus level in different life stages (A) and tissue samples (B) of the black soldier fly: Larvae fed with non-sterile (GN) and sterile (GS) feed and the larval haemolymph of GS (LH), the pupal cell pulp (CP), and from the female adults after mating, a wash of the embryo-laying apparatus (WS) and the afterwards placed embryos of the embryo-laying apparatus (EA), embryos collected from the ovary (EO) and the empty female abdomen (FA), embryos collected from a fly cage after forced exposure to adults (EC) and sterilized (ES).

**Fig. S2.** Network analysis of microbial communities in different life stages (A) and tissue samples (B) of the black soldier fly: Larvae fed with non-sterile (GN) and sterile (GS) feed and the larval haemolymph of GS (LH), the pupal cell pulp (CP), and from the female adults after mating, a wash of the egg-laying apparatus (WS) and the afterwards placed eggs of the egg-laying apparatus (EA), eggs collected from the ovary (EO) and the empty female abdomen (FA), eggs collected from a fly cage after forced exposure to adults (EC) and sterilized (ES).

**Fig. S3.** **(a)** Growth performance of black soldier fly larvae freshly hatched from forced oviposited eggs into sterile Eppendorf tubes, fed sterilized feed (chicken feed 40:60 w/v mixed with water) and **(b)** percentage of pupated and dead larvae, and larvae that had not pupated at the time of experiment termination.

**Fig. S4.** **(a)** Growth performance of black soldier fly larvae reared on a sterilized and non-sterilized feed (chicken feed 40:60 w/v mixed with water) and **(b)** percentage of pupated and dead larvae, and larvae that had not pupated at the time of experiment termination.
