# Supplementary material for: Stage-Specific Microbiota Transitions Throughout Black Soldier Fly Ontogeny
Source: Microb Ecol. 2026 Jan 10;89:41. doi: 10.1007/s00248-025-02691-1 (PMC12860825; doi:10.1007/s00248-025-02691-1)
Supplement: Supplementary file 3 [file 248_2025_2691_MOESM3_ESM.docx]

**Stage-specific microbiota transitions throughout black soldier fly ontogeny**

Thomas Klammsteiner^1^, Carina D. Heussler^1^, Katharina T. Stonig^1^, Heribert Insam^2^, Birgit C. Schlick-Steiner^1^, Florian M. Steiner^1^

^1^ Universität Innsbruck, Department of Ecology, 6020 Innsbruck, Austria

^2^ BioTreaT GmbH, Technikerstr. 21d, 6020 Innsbruck, Austria

**Online Resource 3**

**Tab. S1.** PERMANOVA based on Bray- Curtis analysis in different life stages (A) and tissue samples (B) of the black soldier fly: Larvae fed with non-sterile (GN) and sterile (GS) feed and the larval haemolymph of GS (LH), the pupal cell pulp (CP), and from the female adults after mating, a wash of the egg-laying apparatus (WS) and the afterwards placed eggs of the egg-laying apparatus (EA), eggs collected from the ovary (EO) and the empty female abdomen (FA), eggs collected from a fly cage after forced exposure to adults (EC) and sterilized (ES). Significant codes: 0 '***' 0.001 '**' 0.01 '*' 0.05 '.' 0.1 ' ' 1

A)

Df	SumsOfSqs	MeanSqs	F.Model	R2	Pr(>F)

```
## Stage      3    	3.9545 	1.31815  	12.676 	0.60335 	0.000999 ***
## Residuals 25    	2.5997 	0.10399         	0.39665
## Total     28    	6.5541                 		1.00000
```

B)

Df	SumsOfSqs	MeanSqs	F.Model	R2	Pr(>F)

```
## Tissue     	4    3.6108 	0.90270  	7.3607 	0.55092 	0.000999 ***
## Residuals 	24   2.9433 	0.12264         	0.44908
## Total     	28   6.5541                 		1.00000
```

**Tab. S2.** Pairwise PERMANOVA analysis with Bonferroni correction in different life stages (A) and tissue samples (B) of the black soldier fly: Larvae fed with non-sterile (GN) and sterile (GS) feed and the larval haemolymph of GS (LH), the pupal cell pulp (CP), and from the female adults after mating, a wash of the egg-laying apparatus (WS) and the afterwards placed eggs of the egg-laying apparatus (EA), eggs collected from the ovary (EO) and the empty female abdomen (FA), eggs collected from a fly cage after forced exposure to adults (EC) and sterilized (ES). Significant codes: 0 '***' 0.001 '**' 0.01 '*' 0.05 '.' 0.1 ' ' 1

A)	Larva	Pupa	Adult

Pupa	0.042	-	-

Adult	0.012	0.156	-

Eggs	0.006	0.048	0.006

B)	Eggs	Gut	Haemolymph	Ovarium

Gut	0.02	-	-	-

Haemolymph	0.01	0.04	-	-

Ovarium	0.16	0.15	0.52	-

Ovipositor	0.06	0.03	1.00	0.87

**Tab. S3.** Sanger sequencing and nucleotide BLAST® search results of the isolates gained during the analysis of single individual egg cytoplasm of BSF.

| Isolate | Sequence | NCBI Hit |
| --- | --- | --- |
| 1 | CCAATCCAAAGCCAAACCTGAGAGGGTGATCGGCCACACTGGGACTGAGACACGGCCCAGACTCCTACGGGAGGCAGCAGTAGGGAATCTTCCGCAATGGACGAAAGTCTGACGGAGCAACGCCGCGTGAGTGATGAAGGCTTTCGGGTCGTAAAACTCTGTTGTTAGGGAAGAACAAGTACGAGAGTAACTGCTCGTACCTTGACGGTACCTAACCAGAAAGCCACGGCTAACTACGTGCCAGCAGCCGCGGTAATACGTAGGTGGCAAGCGTTATCCGGAATTATTGGGCGTAAAGCGCGCGCAGGCGGTTTCTTAAGTCTGATGTGAAAGCCCACGGCTCAACCGTGGAGGGTCATTGGAAACTGGGGAACTTGAGTGCAGAAGAGAAAAGCGGAATTCCACGTGTAGCGGTGAAATGCGTAGAGATGTGGAGGAACACCAGTGGCGAAGGCGGCTTTTTGGTCTGTAACTGACGCTGAGGCGCGAAAGCGTGGGGAGCAAACAGGATTAGATACCCTGGTAGTCCACGCCGTAAACGATGAGTGCTAAGTGTTAGAGGGTTTCCGCCCTTTAGTGCTGCAGCTAACGCATTAAGCACTCCGCCTGGGGAGTACGGTCGCAAGACTGAAACTCAAAGGAATTGACGGGGGCCCGCACAAGCGGTGGAGCATGTGGTTTAATTCGAAGCAACGCGAAGAACCTTACCAGGTCTTGACATCCTCTGACAACTCTGGAGATAGAGCGTTCCCCTTCGGGGGACAGAGTGACAGGTGGTGCATGGTTGTCGTCAGCTCGTGTCGTGAGATGTTGGGTTAAGTCCCGCAACGAGCGCAACCCTTGATCTAGTTGCCAGCATTAGTTGGGCACTCTAAGGTGACTGCCGTGACAACCGGAGAAAGGTGGGGATAA | *Bacillus zanthoxyli*  Acc. Nr.: [164882.1](https://www.ncbi.nlm.nih.gov/nucleotide/NR_164882.1?report=genbank&log$=nucltop&blast_rank=1&RID=A28N4KFJ016)  Per. Ident.: 99.33% |
| 2 | CTGCTATACATGCAAGTCGAGCGAACTGATTAGAAGCTTGCTTCTATGACGTTAGCGGCGGACGGGTGAGTAACACGTGGGCAACCTGCCTGTAAGACTGGGATAACTTCGGGAAACCGAAGCTAATACCGGATAGGATCTTCTCCTTCATGGGAGATGATTGAAAGATGGTTTCGGCTATCACTTACAGATGGGCCCGCGGTGCATTAGCTAGTTGGTGAGGTAACGGCTCACCAAGGCAACGATGCATAGCCGACCTGAGAGGGTGATCGGCCACACTGGGACTGAGACACGGCCCAGACTCCTACGGGAGGCAGCAGTAGGGAATCTTCCGCAATGGACGAAAGTCTGACGGAGCAACGCCGCGTGAGTGATGAAGGCTTTCGGGTCGTAAAACTCTGTTGTTAGGGAAGAACAAGTACGAGAGTAACTGCTCGTACCTTGACGGTACCTAACCAGAAAGCCACGGCTAACTACGTGCCAGCAGCCGCGGTAATACGTAGGTGGCAAGCGTTATCCGGAATTATTGGGCGTAAAGCGCGCGCAGGCGGTTTCTTAAGTCTGATGTGAAAGCCCACGGCTCAACCGTGGAGGGTCATTGGAAACTGGGGAACTTGAGTGCAGAAGAGAAAAGCGGAATTCCACGTGTAGCGGTGAAATGCGTAGAGATGTGGAGGAACACCAGTGGCGAAGGCGGCTTTTTGGTCTGTAACTGACGCTGAGGCGCGAAAGCGTGGGGAGCAAACAGGATTAGATACCCTGGTAGTCCACGCCGTAAACGATGAGTGCTAAGTGTTAGAGGGTTTCCGCCCTTTAGTGCTGCAGCTAACGCATTAAGCACTCCGCCTGGGGAGTACGGTCGCAAGACTGAAACTCAAAGGAATTGACGGGGGCCCGCACAAGCGGTGGAGCATGTGGTTTAATTCGAAGCAACGCGAAGAACCTTACCAGGTCTTGACATCCTCTGACAACTCTAGAGATAGAGCGTTCCCCTTCGGGGGACAGAGTGACAGGTGGTGCATGGTTGTCGTCAGCTCGTGTCGTGAGATGTTGGGTTAAGTCCCGCAACGAGCGCAACCTTGATCTTAGTTGCCAGCATTTAGTTGGGCACTCTAAGGTGACTGCCCGTGAC | *Bacillus zanthoxyli*  Acc. Nr.: [164882.1](https://www.ncbi.nlm.nih.gov/nucleotide/NR_164882.1?report=genbank&log$=nucltop&blast_rank=1&RID=A28N4KFJ016)  Per. Ident.: 99.73% |
| 3 | GCTTAACACATGCAAGTCGAACGATGAAGCACCAGCTTGCTGGTGTGGATTAGTGGCGAACGGGTGAGTAACACGTGAGTAACCTGCCCCTCACTCTGGGATAAGCCTGGGAAACTGGGTCTAATACTGGATACGACCGATCTCCGCATGGAGTGTTGGTGGAAAGTTTTTGTGGTGGGGGATGGACTCGCGGCCTATCAGCTTGTTGGTGGGGTAATGGCCTACCAAGGCGACGACGGGTAGCCGGCCTGAGAGGGCGACCGGCCACACTGGGACTGAGACACGGCCCAGACTCCTACGGGAGGCAGCAGTGGGGAATATTGCACAATGGGCGAAAGCCTGATGCAGCGACGCCGCGTGAGGGATGACGGCCTTCGGGTTGTAAACCTCTTTCACCAGGGACGAAGCTAACGTGACGGTACCTGGAGAAGAAGCACCGGCTAACTACGTGCCAGCAGCCGCGGTAATACGTAGGGTGCGAGCGTTGTCCGGAATTATTGGGCGTAAAGAGCTTGTAGGCGGTTTGTCGCGTCTGCTGTGAAAGACCGGGGCTTAACTCCGGTTCTGCAGTGGGTACGGGCAGACTAGAGTGTGGTAGGGGAGACTGGAATTCCTGGTGTAGCGGTGAAATGCGCAGATATCAGGAGGAACACCGATGGCGAAGGCAGGTCTCTGGGCCATTACTGACGCTGAGAAGCGAAAGCATGGGGAGCGAACAGGATTAGATACCCTGGTAGTCCATGCCGTAAACGTTGGGCGCTAGGTGTGGGACTCATTCCACGAGTTCCGTGCCGCAGCTAACGCATTAAGCGCCCCGCCTGGGGAGTACGGCCGCAAGGCTAAAACTCAAAGGAATTGACGGGGGCCCGCACAAGCGGCGGAGCATGCGGATTAATTCGATGCAACGCGAAGAACCTTACCAAGGCTTGACATACACCGGAATCATGCAGAGATGTGTGCGTCTTCGGACTGGTGTACAGGTGGTGCATGGTTGTCGTCAGCTCGTGTCGTGAGATGTTGGGTTAAGTCCCGCAACGAGCGCAACCCTCGTTCCATGTTGCCAGCACTTCGGGTGGGGACTCATGGGAGACTGCCGGGGTCACTCGGAGGAAGGTGGGGATGACGTCAAATCATCATGCCCCTTATGTCTTGGGCTTCCGCATGCTACATGGCCGGGACAGAGGGTTGCGAAACCG | *Dermacoccus nishinomiyaensis*  Acc. Nr.: [044872.1](https://www.ncbi.nlm.nih.gov/nucleotide/NR_044872.1?report=genbank&log$=nucltop&blast_rank=1&RID=A28N4KFJ016)  Per. Ident.: 99.42% |
| 4 | TGCTTAACACATGCAAGTCGAACGATGAAGCACCAGCTTGCTGGTGTGGATTAGTGGCGAACGGGTGAGTAACACGTGAGTAACCTGCCCCTCACTCTGGGATAAGCCTGGGAAACTGGGTCTAATACTGGATACGACCGATCTCCGCATGGAGTGTTGGTGGAAAGTTTTTGTGGTGGGGGATGGACTCGCGGCCTATCAGCTTGTTGGTGGGGTAATGGCCTACCAAGGCGACGACGGGTAGCCGGCCTGAGAGGGCGACCGGCCACACTGGGACTGAGACACGGCCCAGACTCCTACGGGAGGCAGCAGTGGGGAATATTGCACAATGGGCGAAAGCCTGATGCAGCGACGCCGCGTGAGGGATGACGGCCTTCGGGTTGTAAACCTCTTTCACCAGGGACGAAGCTAACGTGACGGTACCTGGAGAAGAAGCACCGGCTAACTACGTGCCAGCAGCCGCGGTAATACGTAGGGTGCGAGCGTTGTCCGGAATTATTGGGCGTAAAGAGCTTGTAGGCGGTTTGTCGCGTCTGCTGTGAAAGACCGGGGCTTAACTCCGGTTCTGCAGTGGGTACGGGCAGACTAGAGTGTGGTAGGGGAGACTGGAATTCCTGGTGTAGCGGTGAAATGCGCAGATATCAGGAGGAACACCGATGGCGAAGGCAGGTCTCTGGGCCATTACTGACGCTGAGAAGCGAAAGCATGGGGAGCGAACAGGATTAGATACCCTGGTAGTCCATGCCGTAAACGTTGGGCGCTAGGTGTGGGACTCATTCCACGAGTTCCGTGCCGCAGCTAACGCATTAAGCGCCCCGCCTGGGGAGTACGGCCGCAAGGCTAAAACTCAAAGGAATTGACGGGGGCCCGCACAAGCGGCGGAGCATGCGGATTAATTCGATGCAACGCGAAGAACCTTACCAAGGCTTGACATACACCGGAATCATGCAGAGATGTGTGCGTCTTCGGACTGGTGTACAGGTGGTGCATGGTTGTCGTCAGCTCGTGTCGTGAGATGTTGGGTTAAGTCCCGCAACGAGCGCAACCCTCGTTCATGTTGCCAGCACTTCGGGTGGGGACTCATGGGAGACTGCCGGGGTCAACTCGGAGGAAGGTGGGGATGACGTCAAATCATCATGCCCCTTATGTCTTGGGCTTCCCGCATGCTACAAG | *Dermacoccus nishinomiyaensis*  Acc. Nr.: [044872.1](https://www.ncbi.nlm.nih.gov/nucleotide/NR_044872.1?report=genbank&log$=nucltop&blast_rank=1&RID=A28N4KFJ016)  Per. Ident.: 99.57% |
| 5 | GGCTGCTTACACATGCAAGTCGAACGATGAAGCACCAGCTTGCTGGTGTGGATTAGTGGCGAACGGGTGAGTAACACGTGAGTAACCTGCCCCTCACTCTGGGATAAGCCTGGGAAACTGGGTCTAATACTGGATACGACCGATCTCCGCATGGAGTGTTGGTGGAAAGTTTTTGTGGTGGGGGATGGACTCGCGGCCTATCAGCTTGTTGGTGGGGTAATGGCCTACCAAGGCGACGACGGGTAGCCGGCCTGAGAGGGCGACCGGCCACACTGGGACTGAGACACGGCCCAGACTCCTACGGGAGGCAGCAGTGGGGAATATTGCACAATGGGCGAAAGCCTGATGCAGCGACGCCGCGTGAGGGATGACGGCCTTCGGGTTGTAAACCTCTTTCACCAGGGACGAAGCTAACGTGACGGTACCTGGAGAAGAAGCACCGGCTAACTACGTGCCAGCAGCCGCGGTAATACGTAGGGTGCGAGCGTTGTCCGGAATTATTGGGCGTAAAGAGCTTGTAGGCGGTTTGTCGCGTCTGCTGTGAAAGACCGGGGCTTAACTCCGGTTCTGCAGTGGGTACGGGCAGACTAGAGTGTGGTAGGGGAGACTGGAATTCCTGGTGTAGCGGTGAAATGCGCAGATATCAGGAGGAACACCGATGGCGAAGGCAGGTCTCTGGGCCATTACTGACGCTGAGAAGCGAAAGCATGGGGAGCGAACAGGATTAGATACCCTGGTAGTCCATGCCGTAAACGTTGGGCGCTAGGTGTGGGACTCATTCCACGAGTTCCGTGCCGCAGCTAACGCATTAAGCGCCCCGCCTGGGGAGTACGGCCGCAAGGCTAAAACTCAAAGGAATTGACGGGGGCCCGCACAAGCGGCGGAGCATGCGGATTAATTCGATGCAACGCGAAGAACCTTACCAAGGCTTGACATACACCGGAATCATGCAGAGATGTGTGCGTCTTCGGACTGGTGTACAGGTGGTGCATGGTTGTCGTCAGCTCGTGTCGTGAGATGTTGGGTTAAGTCCCGCAACGAGCGCAACCCTCGTTCATGTTGCCAGCACTTCGGGTGGGGACTCATGGGAGACTGCCGGGGTCAACTCGGAGGAAGGTGGGGATGACGTCAAATCATCATGCCCCTTATGTCTTGGGCTTCCGCATGCTACAATGGCCGGTACAGAGGGTTGCGAAACCGTGA | *Dermacoccus nishinomiyaensis*  Acc. Nr.: [044872.1](https://www.ncbi.nlm.nih.gov/nucleotide/NR_044872.1?report=genbank&log$=nucltop&blast_rank=1&RID=A28N4KFJ016)  Per. Ident.: 99.42% |
