# Supplementary material for: Stage-Specific Microbiota Transitions Throughout Black Soldier Fly Ontogeny
Source: Microb Ecol. 2026 Jan 10;89(1):41. doi: 10.1007/s00248-025-02691-1 (PMC12860825; doi:10.1007/s00248-025-02691-1)
Supplement: Supplementary file 3 — Supplementary Material 3 [file 248_2025_2691_MOESM3_ESM.docx]

**Stage-specific microbiota transitions throughout black soldier fly ontogeny**

Thomas Klammsteiner^1^, Carina D. Heussler^1^, Katharina T. Stonig^1^, Heribert Insam^2^, Birgit C. Schlick-Steiner^1^, Florian M. Steiner^1^

^1^ Universität Innsbruck, Department of Ecology, 6020 Innsbruck, Austria

^2^ BioTreaT GmbH, Technikerstr. 21d, 6020 Innsbruck, Austria

**Online Resource 3**

**Tab. S1.** PERMANOVA based on Bray- Curtis analysis in different life stages (A) and tissue samples (B) of the black soldier fly: Larvae fed with non-sterile (GN) and sterile (GS) feed and the larval haemolymph of GS (LH), the pupal cell pulp (CP), and from the female adults after mating, a wash of the egg-laying apparatus (WS) and the afterwards placed eggs of the egg-laying apparatus (EA), eggs collected from the ovary (EO) and the empty female abdomen (FA), eggs collected from a fly cage after forced exposure to adults (EC) and sterilized (ES). Significant codes: 0 '***' 0.001 '**' 0.01 '*' 0.05 '.' 0.1 ' ' 1

A)

Df SumsOfSqs MeanSqs F.Model R2 Pr(>F)

## Stage 3 3.9545 1.31815 12.676 0.60335 0.000999 ***

## Residuals 25 2.5997 0.10399 0.39665

## Total 28 6.5541 1.00000

B)

Df SumsOfSqs MeanSqs F.Model R2 Pr(>F)

## Tissue 4 3.6108 0.90270 7.3607 0.55092 0.000999 ***

## Residuals 24 2.9433 0.12264 0.44908

## Total 28 6.5541 1.00000

**Tab. S2.** Pairwise PERMANOVA analysis with Bonferroni correction in different life stages (A) and tissue samples (B) of the black soldier fly: Larvae fed with non-sterile (GN) and sterile (GS) feed and the larval haemolymph of GS (LH), the pupal cell pulp (CP), and from the female adults after mating, a wash of the egg-laying apparatus (WS) and the afterwards placed eggs of the egg-laying apparatus (EA), eggs collected from the ovary (EO) and the empty female abdomen (FA), eggs collected from a fly cage after forced exposure to adults (EC) and sterilized (ES). Significant codes: 0 '***' 0.001 '**' 0.01 '*' 0.05 '.' 0.1 ' ' 1

A) Larva Pupa Adult

Pupa 0.042 - -

Adult 0.012 0.156 -

Eggs 0.006 0.048 0.006

B) Eggs Gut Haemolymph Ovarium

Gut 0.02 - - -

Haemolymph 0.01 0.04 - -

Ovarium 0.16 0.15 0.52 -

Ovipositor 0.06 0.03 1.00 0.87

**Tab. S3.** Sanger sequencing and nucleotide BLAST® search results of the isolates gained during the analysis of single individual egg cytoplasm of BSF.

| Isolate | Sequence | NCBI Hit |
| --- | --- | --- |
| 1 | CCAATCCAAAGCCAAACCTGAGAGGGTGATCGGCCACACTGGGACTGAGACACGGCCCAGACTCCTACGGGAGGCAGCAGTAGGGAATCTTCCGCAATGGACGAAAGTCTGACGGAGCAACGCCGCGTGAGTGATGAAGGCTTTCGGGTCGTAAAACTCTGTTGTTAGGGAAGAACAAGTACGAGAGTAACTGCTCGTACCTTGACGGTACCTAACCAGAAAGCCACGGCTAACTACGTGCCAGCAGCCGCGGTAATACGTAGGTGGCAAGCGTTATCCGGAATTATTGGGCGTAAAGCGCGCGCAGGCGGTTTCTTAAGTCTGATGTGAAAGCCCACGGCTCAACCGTGGAGGGTCATTGGAAACTGGGGAACTTGAGTGCAGAAGAGAAAAGCGGAATTCCACGTGTAGCGGTGAAATGCGTAGAGATGTGGAGGAACACCAGTGGCGAAGGCGGCTTTTTGGTCTGTAACTGACGCTGAGGCGCGAAAGCGTGGGGAGCAAACAGGATTAGATACCCTGGTAGTCCACGCCGTAAACGATGAGTGCTAAGTGTTAGAGGGTTTCCGCCCTTTAGTGCTGCAGCTAACGCATTAAGCACTCCGCCTGGGGAGTACGGTCGCAAGACTGAAACTCAAAGGAATTGACGGGGGCCCGCACAAGCGGTGGAGCATGTGGTTTAATTCGAAGCAACGCGAAGAACCTTACCAGGTCTTGACATCCTCTGACAACTCTGGAGATAGAGCGTTCCCCTTCGGGGGACAGAGTGACAGGTGGTGCATGGTTGTCGTCAGCTCGTGTCGTGAGATGTTGGGTTAAGTCCCGCAACGAGCGCAACCCTTGATCTAGTTGCCAGCATTAGTTGGGCACTCTAAGGTGACTGCCGTGACAACCGGAGAAAGGTGGGGATAA | *Bacillus zanthoxyli*  Acc. Nr.: [164882.1](https://www.ncbi.nlm.nih.gov/nucleotide/NR_164882.1?report=genbank&log$=nucltop&blast_rank=1&RID=A28N4KFJ016)  Per. Ident.: 99.33% |
| 2 | CTGCTATACATGCAAGTCGAGCGAACTGATTAGAAGCTTGCTTCTATGACGTTAGCGGCGGACGGGTGAGTAACACGTGGGCAACCTGCCTGTAAGACTGGGATAACTTCGGGAAACCGAAGCTAATACCGGATAGGATCTTCTCCTTCATGGGAGATGATTGAAAGATGGTTTCGGCTATCACTTACAGATGGGCCCGCGGTGCATTAGCTAGTTGGTGAGGTAACGGCTCACCAAGGCAACGATGCATAGCCGACCTGAGAGGGTGATCGGCCACACTGGGACTGAGACACGGCCCAGACTCCTACGGGAGGCAGCAGTAGGGAATCTTCCGCAATGGACGAAAGTCTGACGGAGCAACGCCGCGTGAGTGATGAAGGCTTTCGGGTCGTAAAACTCTGTTGTTAGGGAAGAACAAGTACGAGAGTAACTGCTCGTACCTTGACGGTACCTAACCAGAAAGCCACGGCTAACTACGTGCCAGCAGCCGCGGTAATACGTAGGTGGCAAGCGTTATCCGGAATTATTGGGCGTAAAGCGCGCGCAGGCGGTTTCTTAAGTCTGATGTGAAAGCCCACGGCTCAACCGTGGAGGGTCATTGGAAACTGGGGAACTTGAGTGCAGAAGAGAAAAGCGGAATTCCACGTGTAGCGGTGAAATGCGTAGAGATGTGGAGGAACACCAGTGGCGAAGGCGGCTTTTTGGTCTGTAACTGACGCTGAGGCGCGAAAGCGTGGGGAGCAAACAGGATTAGATACCCTGGTAGTCCACGCCGTAAACGATGAGTGCTAAGTGTTAGAGGGTTTCCGCCCTTTAGTGCTGCAGCTAACGCATTAAGCACTCCGCCTGGGGAGTACGGTCGCAAGACTGAAACTCAAAGGAATTGACGGGGGCCCGCACAAGCGGTGGAGCATGTGGTTTAATTCGAAGCAACGCGAAGAACCTTACCAGGTCTTGACATCCTCTGACAACTCTAGAGATAGAGCGTTCCCCTTCGGGGGACAGAGTGACAGGTGGTGCATGGTTGTCGTCAGCTCGTGTCGTGAGATGTTGGGTTAAGTCCCGCAACGAGCGCAACCTTGATCTTAGTTGCCAGCATTTAGTTGGGCACTCTAAGGTGACTGCCCGTGAC | *Bacillus zanthoxyli*  Acc. Nr.: [164882.1](https://www.ncbi.nlm.nih.gov/nucleotide/NR_164882.1?report=genbank&log$=nucltop&blast_rank=1&RID=A28N4KFJ016)  Per. Ident.: 99.73% |
| 3 | GCTTAACACATGCAAGTCGAACGATGAAGCACCAGCTTGCTGGTGTGGATTAGTGGCGAACGGGTGAGTAACACGTGAGTAACCTGCCCCTCACTCTGGGATAAGCCTGGGAAACTGGGTCTAATACTGGATACGACCGATCTCCGCATGGAGTGTTGGTGGAAAGTTTTTGTGGTGGGGGATGGACTCGCGGCCTATCAGCTTGTTGGTGGGGTAATGGCCTACCAAGGCGACGACGGGTAGCCGGCCTGAGAGGGCGACCGGCCACACTGGGACTGAGACACGGCCCAGACTCCTACGGGAGGCAGCAGTGGGGAATATTGCACAATGGGCGAAAGCCTGATGCAGCGACGCCGCGTGAGGGATGACGGCCTTCGGGTTGTAAACCTCTTTCACCAGGGACGAAGCTAACGTGACGGTACCTGGAGAAGAAGCACCGGCTAACTACGTGCCAGCAGCCGCGGTAATACGTAGGGTGCGAGCGTTGTCCGGAATTATTGGGCGTAAAGAGCTTGTAGGCGGTTTGTCGCGTCTGCTGTGAAAGACCGGGGCTTAACTCCGGTTCTGCAGTGGGTACGGGCAGACTAGAGTGTGGTAGGGGAGACTGGAATTCCTGGTGTAGCGGTGAAATGCGCAGATATCAGGAGGAACACCGATGGCGAAGGCAGGTCTCTGGGCCATTACTGACGCTGAGAAGCGAAAGCATGGGGAGCGAACAGGATTAGATACCCTGGTAGTCCATGCCGTAAACGTTGGGCGCTAGGTGTGGGACTCATTCCACGAGTTCCGTGCCGCAGCTAACGCATTAAGCGCCCCGCCTGGGGAGTACGGCCGCAAGGCTAAAACTCAAAGGAATTGACGGGGGCCCGCACAAGCGGCGGAGCATGCGGATTAATTCGATGCAACGCGAAGAACCTTACCAAGGCTTGACATACACCGGAATCATGCAGAGATGTGTGCGTCTTCGGACTGGTGTACAGGTGGTGCATGGTTGTCGTCAGCTCGTGTCGTGAGATGTTGGGTTAAGTCCCGCAACGAGCGCAACCCTCGTTCCATGTTGCCAGCACTTCGGGTGGGGACTCATGGGAGACTGCCGGGGTCACTCGGAGGAAGGTGGGGATGACGTCAAATCATCATGCCCCTTATGTCTTGGGCTTCCGCATGCTACATGGCCGGGACAGAGGGTTGCGAAACCG | *Dermacoccus nishinomiyaensis*  Acc. Nr.: [044872.1](https://www.ncbi.nlm.nih.gov/nucleotide/NR_044872.1?report=genbank&log$=nucltop&blast_rank=1&RID=A28N4KFJ016)  Per. Ident.: 99.42% |
| 4 | TGCTTAACACATGCAAGTCGAACGATGAAGCACCAGCTTGCTGGTGTGGATTAGTGGCGAACGGGTGAGTAACACGTGAGTAACCTGCCCCTCACTCTGGGATAAGCCTGGGAAACTGGGTCTAATACTGGATACGACCGATCTCCGCATGGAGTGTTGGTGGAAAGTTTTTGTGGTGGGGGATGGACTCGCGGCCTATCAGCTTGTTGGTGGGGTAATGGCCTACCAAGGCGACGACGGGTAGCCGGCCTGAGAGGGCGACCGGCCACACTGGGACTGAGACACGGCCCAGACTCCTACGGGAGGCAGCAGTGGGGAATATTGCACAATGGGCGAAAGCCTGATGCAGCGACGCCGCGTGAGGGATGACGGCCTTCGGGTTGTAAACCTCTTTCACCAGGGACGAAGCTAACGTGACGGTACCTGGAGAAGAAGCACCGGCTAACTACGTGCCAGCAGCCGCGGTAATACGTAGGGTGCGAGCGTTGTCCGGAATTATTGGGCGTAAAGAGCTTGTAGGCGGTTTGTCGCGTCTGCTGTGAAAGACCGGGGCTTAACTCCGGTTCTGCAGTGGGTACGGGCAGACTAGAGTGTGGTAGGGGAGACTGGAATTCCTGGTGTAGCGGTGAAATGCGCAGATATCAGGAGGAACACCGATGGCGAAGGCAGGTCTCTGGGCCATTACTGACGCTGAGAAGCGAAAGCATGGGGAGCGAACAGGATTAGATACCCTGGTAGTCCATGCCGTAAACGTTGGGCGCTAGGTGTGGGACTCATTCCACGAGTTCCGTGCCGCAGCTAACGCATTAAGCGCCCCGCCTGGGGAGTACGGCCGCAAGGCTAAAACTCAAAGGAATTGACGGGGGCCCGCACAAGCGGCGGAGCATGCGGATTAATTCGATGCAACGCGAAGAACCTTACCAAGGCTTGACATACACCGGAATCATGCAGAGATGTGTGCGTCTTCGGACTGGTGTACAGGTGGTGCATGGTTGTCGTCAGCTCGTGTCGTGAGATGTTGGGTTAAGTCCCGCAACGAGCGCAACCCTCGTTCATGTTGCCAGCACTTCGGGTGGGGACTCATGGGAGACTGCCGGGGTCAACTCGGAGGAAGGTGGGGATGACGTCAAATCATCATGCCCCTTATGTCTTGGGCTTCCCGCATGCTACAAG | *Dermacoccus nishinomiyaensis*  Acc. Nr.: [044872.1](https://www.ncbi.nlm.nih.gov/nucleotide/NR_044872.1?report=genbank&log$=nucltop&blast_rank=1&RID=A28N4KFJ016)  Per. Ident.: 99.57% |
| 5 | GGCTGCTTACACATGCAAGTCGAACGATGAAGCACCAGCTTGCTGGTGTGGATTAGTGGCGAACGGGTGAGTAACACGTGAGTAACCTGCCCCTCACTCTGGGATAAGCCTGGGAAACTGGGTCTAATACTGGATACGACCGATCTCCGCATGGAGTGTTGGTGGAAAGTTTTTGTGGTGGGGGATGGACTCGCGGCCTATCAGCTTGTTGGTGGGGTAATGGCCTACCAAGGCGACGACGGGTAGCCGGCCTGAGAGGGCGACCGGCCACACTGGGACTGAGACACGGCCCAGACTCCTACGGGAGGCAGCAGTGGGGAATATTGCACAATGGGCGAAAGCCTGATGCAGCGACGCCGCGTGAGGGATGACGGCCTTCGGGTTGTAAACCTCTTTCACCAGGGACGAAGCTAACGTGACGGTACCTGGAGAAGAAGCACCGGCTAACTACGTGCCAGCAGCCGCGGTAATACGTAGGGTGCGAGCGTTGTCCGGAATTATTGGGCGTAAAGAGCTTGTAGGCGGTTTGTCGCGTCTGCTGTGAAAGACCGGGGCTTAACTCCGGTTCTGCAGTGGGTACGGGCAGACTAGAGTGTGGTAGGGGAGACTGGAATTCCTGGTGTAGCGGTGAAATGCGCAGATATCAGGAGGAACACCGATGGCGAAGGCAGGTCTCTGGGCCATTACTGACGCTGAGAAGCGAAAGCATGGGGAGCGAACAGGATTAGATACCCTGGTAGTCCATGCCGTAAACGTTGGGCGCTAGGTGTGGGACTCATTCCACGAGTTCCGTGCCGCAGCTAACGCATTAAGCGCCCCGCCTGGGGAGTACGGCCGCAAGGCTAAAACTCAAAGGAATTGACGGGGGCCCGCACAAGCGGCGGAGCATGCGGATTAATTCGATGCAACGCGAAGAACCTTACCAAGGCTTGACATACACCGGAATCATGCAGAGATGTGTGCGTCTTCGGACTGGTGTACAGGTGGTGCATGGTTGTCGTCAGCTCGTGTCGTGAGATGTTGGGTTAAGTCCCGCAACGAGCGCAACCCTCGTTCATGTTGCCAGCACTTCGGGTGGGGACTCATGGGAGACTGCCGGGGTCAACTCGGAGGAAGGTGGGGATGACGTCAAATCATCATGCCCCTTATGTCTTGGGCTTCCGCATGCTACAATGGCCGGTACAGAGGGTTGCGAAACCGTGA | *Dermacoccus nishinomiyaensis*  Acc. Nr.: [044872.1](https://www.ncbi.nlm.nih.gov/nucleotide/NR_044872.1?report=genbank&log$=nucltop&blast_rank=1&RID=A28N4KFJ016)  Per. Ident.: 99.42% |
